# Post-marital residence patterns and the timing of reproduction: evidence from a matrilineal society

**DOI:** 10.1098/rspb.2023.0159

**Published:** 2023-03-29

**Authors:** Juan Du, Yaming Huang, Peng-Peng Bai, Liqiong Zhou, Sarah Myers, Abigail E. Page, Ruth Mace

**Affiliations:** ^1^ State Key Laboratory of Grassland Agro-ecosystem, College of Ecology, Lanzhou University, Lanzhou, People's Republic of China; ^2^ Department of Anthropology, University College London, London, UK; ^3^ BirthRites Independent Max Planck Research Group, Max Planck Institute for Evolutionary Anthropology, Leipzig, Germany; ^4^ Department of Population Health, London School of Hygiene and Tropical Medicine, London, UK

**Keywords:** post-marital residence, age at first birth, age at last birth, reproductive success

## Abstract

Humans exhibit a broad range of post-marital residence patterns and there is growing recognition that post-marital residence predicts women's reproductive success; however, the nature of the relationship is probably dependent on whether co-resident kin are cooperators or competitors. Here, we explore this relationship in a Tibetan population, where couples practice a mixture of post-marital residence patterns, co-residing in the same village with the wife's parents, the husband's parents or endogamously with both sets of parents. Using detailed demographic data from 17 villages we find that women who live with only their own parents have an earlier age at first birth (AFB) and age at last birth (ALB) than women who live with only their parents-in-law. Women who co-reside with both sets of parents have the earliest AFB and ALB. However, those with co-resident older siblings postponed reproduction, suggestive of competition-related delay. Shifts to earlier reproductive timing were also observed in relation to the imposition of family planning policies, in line with Fisherian expectations. Our study provides evidence of the costs and benefits to women's direct fitness of co-residing with different kin, against a backdrop of adaptive responses to cultural constraints on completed fertility.

## 1. Introduction

Humans exhibit a strong tendency toward exogamy, female dispersal and male philopatry (i.e. virilocal residence) [1], but there is nevertheless significant diversity across and within cultures. Previous research has indicated that residence patterns covary with socio-ecological factors such as warfare [2–4], migration [5], religion [6], subsistence [7,8] and communal breeding [9]. Virilocal residence is often associated with the presence of movable property, for example, domestic animals in pastoralist societies [3,8]; whereas uxorilocal residence (i.e. with the wife's family) and matriliny often correlates with horticulture and a lack of movable wealth [8,10–13]. Such variation is likely to be associated with differing reproductive strategies among women as residence structures whether maternal or paternal kin are present to either cooperate or compete with, thus increasing or decreasing access to the resources necessary for reproduction [14]. Here we explore the relationship between post-marital co-residence and three different measures of reproductive timing among Tibetans in Yunnan province, who display a mixed residence pattern, using demographic data spanning over 50 years.

Completed family size and age-specific fertility are commonly used as measures of reproductive success; however, China's implementation of family planning policies in the late 1970s, capping women's reproductive output, may make such proxies less useful. Nevertheless, the timing of reproductive events across the life course has important implications for individual fitness, becoming more salient as fertility rates decline globally [11]. In this field site, urban Tibetans were limited to two children in 1983 and rural Tibetans to three in 1984 [15]. Limits were initially patchily enforced before becoming strictly so in the early 1990s. Where completed fertility is extrinsically constrained in such a manner, the timing of reproductive decisions are useful proxies for reproductive success because they are unlikely to reflect downstream consequences of decisions regarding completed fertility [11]. Here we examine variation in age at first birth (AFB), interbirth intervals (IBI), and age at last birth (ALB) by post-marital co-residence with parents and siblings in a context of rapidly changing fertility trends in response to top-down fertility policies.

Co-residence patterns are of interest because work on cooperative childrearing has extensively argued that allomothers (any individual other than the mother who invests time or energy into a child and/or their mother) add to maternal resources (via the direct action of childcare or indirect actions of investment in the mother, provisioning or household tasks) [16–22]. As a result, a mother's resources increase, lessening the trade-off between the quantity and ‘quality’ of offspring and potentially resulting in increased reproductive output [23–25]. With reference to reproductive timing, we expect allomothering to be associated with earlier AFB, later ALB and shorter IBIs as the costs of reproduction are reduced, allowing mothers to increase their fertility without experiencing a depression in child survival and/or condition [23,26]. Accordingly, Turke [27] found in the Ifaluk of Melaneisa families with first born daughters experienced later ALB and increased reproductive success, matching results found elsewhere [28–30]. These studies suggest that early born siblings are ‘helpers-at-the-nest’, providing childcare for younger siblings before they can successfully invest in their own reproductive effort, boosting parental reproductive fitness [31]. Beyond offspring assistance, Mathews & Sear [32,33] have demonstrated in a British sample that childcare support by other relatives increased the likelihood of progression to a second birth, and regularly seeing a higher number of emotionally supportive relatives increased the risk of having a first child. Likewise, in eighteenth- to twentieth-century Poland the absence of various grandparents was associated with decreased risk of parity progression, ranging between 16% and 40% [34].

Overall, co-residence with kin—assuming they are engaged in cooperative childrearing—is expected to promote the mother's and/or her children's fitness. The lineage of the kin, however, matters. There is a consistent finding of a matrilineal bias in both the level of allomaternal investments and the consequences of this investment (or proxies of it) [35–38]. There are several evolutionary-based hypotheses to explain this trend, considering the identical coefficients of relatedness (assuming little paternity uncertainty) between maternal and paternal grandparents with their grandchildren (*r* = 0.25), but the different degrees of relatedness with their mother (maternal grandparents *r* = 0.5 versus paternal grandparents *r* = 0). This matters as allomaternal investments do not necessarily promote the fitness of the child directly but may also act indirectly by promoting the fitness of the mother [16,39]. A mother may re-invest this saved energy back into a child, resulting in ultimately improved child outcomes [16,17,39,40,41]. Alternatively, she may invest the additional resources in her own soma, future reproduction (with another partner), or in cooperative activities with other matrilineal kin [35]. In this case, the inclusive fitness benefits of maternal kin care are unchanged, while those of paternal kin are uncertain. Therefore, while paternal kin do help, all else being equal, we expect maternal kin to do more and be associated with greater positive outcomes than paternal kin. In line with this, in eighteenth- and nineteenth-century Finland and Canada [42,43] maternal grandparents' presence was associated with earlier ages of first birth, as were maternal grandmothers in the matrilineal Mosuo [21], while in Italy [44] and Germany [45] they were associated with an increased likelihood of having a first child, and with increased grandchild survival in pre-industrial Finland [43], while paternal grandparents were not.

Reviewing the literature, however, highlights that the relationship between fertility and kinship lineage is not this clear cut. In some cases, both grandparents are positively associated with fertility outcomes [46,47]. In other cases, paternal kin are positively associated with fertility, while the mother's parents were more often associated with anti-natal effects [25], as shown in the Gambia [48] and in India [49] which may be understood in terms of maternal kin indirectly enhancing child ‘quality’ by protecting the mother from reproductive depletion, while paternal kin favour quantity. In the Mosuo of China the age at first birth was reduced in patrilineal settings [50], while in Thailand co-residence with the husband's kin was associated with shorter IBIs [51], ultimately increasing the number of live births, a finding mirrored in the Gambia [48] and eighteenth-century Germany [52].

However, on the other side of the coin from cooperation are competition and conflict. Cooperation, by definition, is a behaviour which has evolved due to the benefit to the recipient [53], causing the giver to suffer some short-term costs. Tension can occur then when the giver's costs are high, or the perceived benefits low, causing conflict [54]. In the case of allomothering, intergenerational conflict may occur when parents want to use the support of their children, ultimately delaying the children's age at first birth [54]. This appears particularly the case in resource-scarce areas, as local resource competition for reproduction is increased [55]. In a large cross-cultural study parental presence was found to be associated with later age of first birth, indicative of conflict [54]. Arguably, from an indirect fitness perspective, as a sibling's stake in their siblings (assuming they are full siblings) is equal to that of their own children, parents are expected to win such intergenerational conflicts because their fitness benefits are reduced when raising a grandchild, as compared to their own child [54]. Therefore, within a system of cooperative childrearing, co-residence is sometimes associated with later AFB, particularly in matrilocal settings (given the theoretical assumption of increased indirect benefits associated with cooperation) in line with evidence reviewed by Sear & Coall [25].

This conflictual relationship may also be visible specifically in relation to which siblings are present in the household. For instance, elder brothers in the Gambia were associated with a reduced probability of sisters giving birth [48], a pattern also observed in Aché hunter–gatherers [56]. This may be the result of younger sisters supporting their brother's reproduction, at a cost to their own [48]. Whether siblings are older or younger appears to matter, as in historic Poland younger sisters were positively associated with completed fertility of their older siblings, while overall number of siblings were negatively correlated with completed family sizes [34]. Similar patterns were found in preindustrial Finland, where same-sex elder siblings’ presence was associated with reduced reproductive success in the focal individual [57]. In the matrilineal (mostly duolocal) Mosuo, co-resident aunts and sisters were associated with slower reproduction indicating competition between same-sex relatives in the same household [58]. Competition between brothers has also been demonstrated in patrilineal Amdo Tibetans, with increased wealth and earlier ages at first birth for women married to men with no brothers or non-reproductive brothers who became celibate monks [59,60]. Ultimately, exploring co-residence with parents and siblings is important to better understand reproductive timing.

Cooperative versus competitive dynamics are also potentially impacted by the relatedness of an individual to the group, which varies across the life-course as a result of sex-based dispersal [61]. When females disperse at marriage, they initially have a lower genetic relatedness to the group they relocate to than their mother-in-law, increasing the indirect fitness costs of forgoing their own reproduction to help others. However, as time goes on and they reproduce, the average local relatedness increases, decreasing the costs and increasing the benefits of cooperation; in such circumstances, we might expect earlier ALB as a result of mothers-in-law ceding competition to their daughters-in-law [11]. In line with this prediction, Mattison *et al.* [11,22] found earlier ALB in patrilineal, patrilocal Mosuo villages, as compared to matrilineal, matrilocal ones, *despite* Chinese family planning policies constraining shifts in the degree of relatedness across the lifecourse. However, the authors highlight that the Mosuo, like the current study population, have low levels of fertility, reducing the degree of reproductive overlap which is hypothesized to drive changes in reproductive timing. Furthermore**,** Snopkowski & Sear [46] in an Indonesian study and Yang *et al.* [62] in western China found no evidence of earlier ages of ALB in patrilocal settings. Given this inconsistency, we expect cooperative dynamics to be better predictors of reproductive timing than group relatedness, but nonetheless siblings remain a key source of conflict.

Finally, China's policy of limiting the number of children women could have also has potential implications for the fitness consequences of reproductive timing. Where quality–quantity trade-offs are weak, as is likely the case when ‘quantity’ is not an option, Fisher's [63] notion of reproductive value comes to the fore. Parental reproductive success gains greater marginal benefits from earlier- versus later-born offspring [64], in addition to which future discounting favours earlier-born offspring with sufficient interbirth intervals. Earlier bouts of reproduction decrease generation times and earlier-born individuals make up a relatively higher proportion of the gene pool [65,66]. Conversely, delaying reproduction increases the chances of maternal mortality prior to both the birth and maturation of her subsequent child [67]. This means that reproductive timing may be as important an element of fitness as total reproductive output. In growing populations such as that in our field site over the last five decades, earlier reproduction should be favoured; the extent to which this is irrespective of residence pattern, would be expected to be determined by the degree to which quality–quantity trade-offs were constrained by the two-three child limit imposed on Tibetans. Furthermore, the need to limit births may reduce ‘wear and tear’ on the mother and thus reduce the need to pace births; in other words, the fertility mortality trade-off is relaxed. Finally, individuals may fear that more restrictive government family planning policies could emerge in future, accelerating births now given future uncertainty.

This research was conducted in a matrilineal society, where marriages may be exogamous (i.e. from beyond the natal village) or endogamous (i.e. from within the natal village), and a range of post-marital residence patterns are practiced likely structuring the levels of cooperation and competition women experience after marriage. An overview of our predictions regarding how the timing of a woman's ABF, IBI and ALB relate to her scenario, in terms of post-marital residence pattern and reproductive career overlap with family planning policy implementation, can be seen in table 1. The predictions outlined are based on the following rationale: Women who co-reside with their own parents often inherit the most family resources and have easier access to matrilineal kin and by extension allomothers—as a result we expect they will have earlier AFBs, later ALBs and shorter IBIs than those dispersing. Women who live in the same village as both their parents and parents-in-law will both inherit more than dispersing women and likely have access to a wider range of kin and so the greatest allomaternal investments—as a result they will have the earliest AFBs, latest ALBs and shortest IBIs of all residence patterns. While women who disperse from their natal village and co-reside with their husband's parents will get the least help from kin—as a result they will have the latest AFB, earliest ALB, and longest IBIs. Given their potentially cooperative or competitive dynamics, we make a range of competing predictions regarding co-residence with siblings: Co-residence with the wife's siblings may increase resource competition (i)—as a result living with any siblings will increase AFB, increase IBIs and bring forward ALB. Conversely, all siblings may represent a source of allomothering (ii)—as a result living with any siblings will be associated with earlier AFB, later ALB and shorter IBIs. Alternatively, younger siblings specifically may act as allomothers for older siblings—as a result living with younger siblings may be associated with earlier AFB, later ALB, and shorter IBIs and living with older siblings the converse. Finally, following Fisherian logic, we predict a shift to earlier reproductive timing in cohorts whose reproductive years fall post-family planning policy implementation, with the shift greater in younger cohorts whose entire reproductive years were under restriction. We explore these scenarios using detailed demographic data from 17 Tibetan villages (677 households and 3836 individuals, of which 1795 were women).

**Table 1.** An overview of predictions regarding the relationship between a woman's scenario and her reproductive timing.

woman's scenario			age at first birth (AFB)	interbirth interval (IBI)	age at last birth (ALB)
co-residence with parents	both parents		earliest	shortest	latest
	own parents		earlier	shorter	later
	husband's parents		latest	longest	earliest
co-residence with siblings	with any siblings	i	later	longer	earlier
		ii	earlier	shorter	later
	with younger siblings		earlier	shorter	later
	with older siblings		later	longer	earlier
reproductive career overlap with family planning policy	no overlap		latest	—	latest
	partial overlap		earlier	—	earlier
	complete overlap		earliest	—	earliest

## 2. Methods

### (a) Study area

Data collection was conducted in Diqing Tibetan autonomous prefecture, Yunnan province, China. The town administers an area covering of nearly 9000 km^2^, with an average altitude of 2830 m [68] and it has three central townships under its jurisdiction [69]. Ethnographic fieldwork was undertaken in 17 villages associated with one township in 2015 and 2021; the 17 villages are geographically close to each other with little social or demographic variation. The area consists of half farming and half pastoral land. Ethnically, there are about 92.4% Tibetans in this area, sharing a Tibetan dialect [69,70]. Behaviour is deeply shaped by the doctrines of Tibetan Buddhism, whose norms stem from the integration of non-local Hinduism with the regional Bon religion [68]. Many adults are illiterate since educational attainment is generally low, but since compulsory education policy was implemented in 2000 children have been receiving a formal education until middle school [71].

While wealth differentials are not large, Tibetans do compete for status via house construction, with luxurious houses associated with respect and individuals who are able to stay with their natal family inherit most of the parental resources. Traditionally, Tibetans had three forms of marriage: polyandry, polygyny, and monogamy [72] but the population now is predominately monogamous [70] and marriages in our sample are all monogamous. Both exogamous and endogamous marriage are common, though parents have power in deciding who their children marry, preferring marriages with natal or neighbouring village members [68,72]. First-born children, irrespective of sex, commonly co-reside with their parents after marriage and inherit parental wealth, whereas later born will either disperse to other villages at marriage or stay in the same village, but not the same house, as their parents. It is very rare to marry someone from outside of the township [68,70].

### (b) Data collection

Demographic data were collected in 2015 and 2021 from 677 households from 17 Tibetan villages, encompassing 3836 individuals (1791 males and 1795 females). J.D., Y.H., L.Z. and P.P.B. with the help of local assistants interviewed every adult man and woman in each household using reproductive and marriage questionnaires.

Survey questions included details of marriage history (marriages, type of marriage and divorces, where they were living in each marriage), birth history (number of children and dates of birth), details of siblings and the current residence location at the village level of parents and siblings. If parents were deceased, we asked where they had lived and marriage history, if alive at the time of marriage. From this sample, we obtained the post-marriage residence location at the time of reproduction relative to the parents of 1299 couples, 428 of whom co-resided in the same village with the wife's parents only, 389 in the same village with the husband's parents only and 482 couples in the same village with both sets of parents (electronic supplementary material, figures S1 and S2). Women's sibling co-residence was categorized in four ways: number of co-resident older brothers, number of co-resident younger brothers, number of co-resident older sisters and number of co-resident younger sisters.

### (c) Sample characteristics

**Figure 1.**
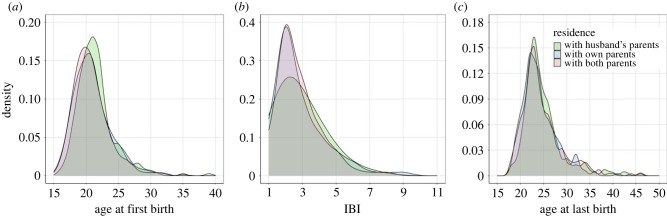
Density plots of (*a*) age at first birth, (*b*) inter-birth interval and (*c*) age at last birth across different post-marital residence patterns. Different colours indicate different post-marital residence patterns: green = with husband's parents, blue = with own parents, red = with both parents.

The average age of the women when surveyed was 46.4 ± 14.4 years. The average age at first birth was 21.0 ± 2.9 and the average age at last birth was 25.3 ± 5.1 years. The average IBI for the entire sample was 2.095 ± 1.603 years (min = 1 years, max = 11 years), which varied little by parity opening the interval (1^st^ IBI = 2.945 ± 1.668; 2^nd^ IBI = 2.693 ± 1.409; 3^rd^ IBI = 3.0 ± 1.603). The trends presented in figure 1 highlight that overall, the distributions between the different patterns of parental co-residence were similar across the three reproductive outcomes, with the exception that the curve for co-residence with husband's parents is less peaked at 2 years for IBIs, and retains slightly higher density at longer IBIs. The peak for AFB is also slightly shifted to the right (to age 22) for co-residence with husband's parents, diverging from age 20 to 21 in co-residence with own or both parents.

### (d) Statistical analysis

Our various predictions regarding the relationships between a woman's reproductive timing and her scenario (table 1) are explored across three models, one for each of our outcomes: AFB, IBI and ALB. Cox proportional-hazards regressions were applied to the time-dependent outcomes AFB and ALB [73] and a multi-level Cox regression model was used for IBI. As the three models share numerous features we discuss them collectively here, while their results are presented in separate sections by outcome below. Discrete time-to-event data were used in all models. Full variable information for each model is given in electronic supplementary material, tables S1–S3. Sample size varies by model due to not all women having experienced a first birth, progressed beyond parity one, or being old enough to be considered to have ceased reproducing; for a breakdown of residence pattern by subset see the results tables.

In all models, we included reproductive cohort of the mother to control for changing fertility policies during the study period: cohorts are separated into 5-year intervals, the earliest acting as the reference category and representing the birth cohort of the oldest women in the sample. As our interest here is on reproduction, these reproductive cohorts represent women's birth cohorts plus 16 years, matching the approach followed by Mattison *et al.* [11]. All models also include a control term for the mother's birth order (standardized permitting comparison of effect size), as earlier born mothers were more likely to remain with their own parents and inherit more wealth than siblings, which may have independent fitness consequences. In the IBI models, given the influence of fertility policies during the study period, while most women had two children (i.e. one interbirth interval, *n* = 861), 196 went on to have a third child and an additional 99 had four or more children. Please see electronic supplementary material, table S4 for a breakdown of IBI length by reproductive cohort. As a result, we conducted a multi-level Cox regression model in which the outcome was the duration of the IBIs which were nested by women as random effects. Regarding ALB, we only included women who had given birth to at least one child. Women were right-censored if they were over 50 years old *or* had an IBI longer than 8 years in line with similar work [11]. Only 16 women (1.38%) have interbirth intervals of longer than 8 years.

We ran all models first with the wife's siblings only and then the addition of the husband's siblings; results were similar across models, but due to missing information the available sample size was reduced when adding the husband's siblings. In the interests of maximizing statistical power, we present the wife's siblings only models here and the expanded models can be found in the SI (see electronic supplementary material, tables S5–S7).

All the statistical analyses were conducted in R (v. 4.0.5) [74] using the packages *survival* [75], *survminer* [76]. All code and data for the analysis can be found at https://osf.io/sakq9.

## 3. Results

### (a) Age at first birth (AFB)

**Figure 2.**
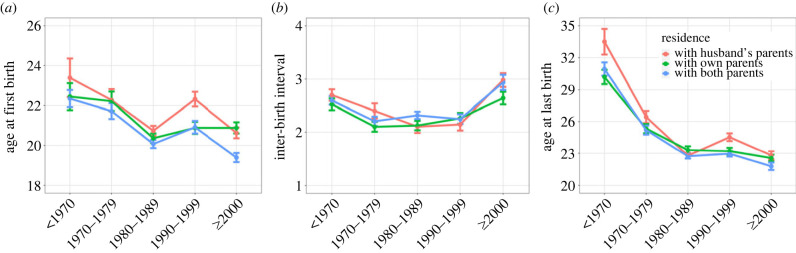
(*a*) Age at first birth, (*b*) inter-birth interval and (*c*) and age at last birth by cohort, which is divided by 10-year intervals reflecting the time of turning 16 years old. Cohorts were classified as time before the implementation of the Chinese fertility policy (less than 1980), intermediate time (1980–1989) and time after the implementation of ‘two-child’ policy (≥1990).

**Figure 3.**
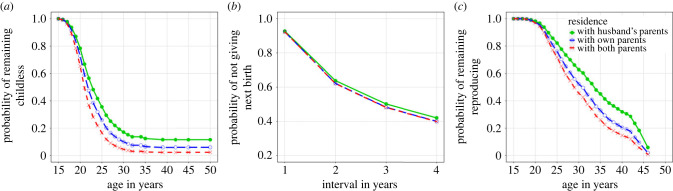
Predicted probability of (*a*) remaining childless, (*b*) not giving next birth and (*c*) remaining reproducing across different post-marital residence patterns. Lines indicate post-marital residence patterns: green filled circle point and solid line = with husband's parents, blue open circle and long dash line = with own parents, red crossing point and dash line = with both parents. Please note all lines are present but overlapping for plot *b*.

The results of our AFB model can be seen in table 2. In line with predictions regarding parental co-residence (table 1), women who resided with their own parents only had earlier AFB than those living with only their husband's parents (HR = 1.306), while those living with both sets of parents had still earlier births (HR = 1.746, figure 3*a*). Regarding our competing predictions relating to co-residence with siblings (table 1), we find an older sibling co-residing in the same village was associated with later AFB, irrespective of sibling gender (HR_brother_ = 0.826; HR_sister_ = 0.770); co-residence with younger siblings, however, showed no such association. In line with predictions regarding reproductive career overlap with family planning policy (table 1), AFB for those turning 16 between 1970 and 79 was earlier than that of women turning 16 prior to 1970, the decline then becomes steeper for those maturing around the time of family planning policy implementation in the early 1980s, then fluctuating in younger cohorts; this was a relatively large effect compared to other predictors in the model. Figure 2 reveals a clear downward trend in AFB regardless of type of co-residence.

**Table 2.** Cox proportional-hazards regression model of afb (*n* = 1000 females with 6269 person-years; having first birth = 1, not having first birth = 0, *n* = 976 events). Statistical significance is indicated in bold. Hazard ratios (HR) above 1.0 indicates earlier AFB.

variables	HR [95% CI]	*p*-value
cohort (ref: <1965)		
1965–1969	1.264 [0.867, 1.843]	0.224
1970–1974	0.988 [0.678, 1.440]	0.952
**1975–1979**	**1.864 [1.309, 2.656]**	**<0**.**001**
**1980–1984**	**2.691 [1.961, 3.694]**	**<0**.**001**
**1985–1989**	**2.302 [1.689, 3.138]**	**<0**.**001**
**1990–1994**	**1.648 [1.213, 2.240]**	**0**.**001**
**1995–1999**	**1.998 [1.430, 2.793]**	**<0**.**001**
**2000–2004**	**2.136 [1.557, 2.930]**	**<0**.**001**
**2005–2009**	**2.743 [1.953, 3.852]**	**<0**.**001**
**≥2010**	**1.614 [1.101, 2.366]**	**0**.**014**
**birth order**	**1.157 [1.044, 1.281]**	**0**.**005**
**older brother present**	**0.826 [0.712, 0.959]**	**0**.**012**
younger brother present	1.008 [0.899, 1.130]	0.893
**older sister present**	**0.770 [0.661, 0.897]**	**<0**.**001**
younger sister present	0.932 [0.819, 1.061]	0.290
residence husband's parents (*n* = 272)	**reference**	
**with own parents (*n* = 361)**	**1.306 [1.063, 1.606]**	**0**.**011**
**with both parents (*n* = 367)**	**1.746 [1.410, 2.161]**	**<0**.**001**

We also found that birth order had an inverse relationship with AFB, as a one standard deviation increase in birth order was associated with an increased hazard of first birth (HR = 1.157); post hoc exploration of this counterintuitive result indicates it is due to controlling for parental and sibling co-residence, as removing these variables from the model reverses the direction of the association (see electronic supplementary material table S9).

### (b) Inter-birth interval (IBI)

The results of our IBI model can be seen in table 3. We find limited evidence of a difference in IBIs by parental co-residence pattern (figure 3*b*) or co-residence with brothers. There is a relationship between co-residence in the same village with older sisters and IBI; it appears women co-residing with older sisters had a decreased HR of progressing to the next birth (HR = 0.878), possibly indicating a delaying effect of allocare provision (table 1), but wide CIs necessitate caution in this interpretation (95% CI [0.760, 1.014]). Temporal effects are again suggested as individuals who turned 16 after 1980 were more likely to have a longer IBIs compared to in reproductive cohorts from 1965 or earlier (table 3). This effect while initially weaker (HR = 0.812) in the 1980–1984 cohort, strengthens throughout time in terms of the effect sizes and strength of evidence, reaching its lowest hazard in the 2005–2009 cohort when women demonstrated a 58.4% reduction in the hazard of having a subsequent birth per year. This is driven by declining ALB increasing final IBIs (which are not closed by a birth over time); figure 2 plots IBIs closed by a birth only, indicating a U-shaped pattern.

**Table 3.** Mixed effect cox model of IBI (*n* = 970 females with 2120 IBI and 6736 person-years; having a next birth = 1, no birth occurring = 0, *n* = 1023 events). Statistical significance is indicated in bold. Hazard ratios (HR) above 1.0 indicates a shorter IBI.

variables	HR [95% CI]	*p*
**fixed effects**		
cohort (ref: <1965)		
1965–1969	1.098 [0.850, 1.418]	0.475
1970–1974	0.991 [0.750, 1.310]	0.952
1975–1979	1.002 [0.771, 1.302]	0.988
1980–1984	0.812 [0.643, 1.027]	0.082
**1985–1989**	**0.776 [0.612, 0.984]**	**0**.**036**
**1990–1994**	**0.665 [0.520, 0.850]**	**0**.**001**
**1995–1999**	**0.643 [0.487, 0.849]**	**0**.**002**
**2000–2004**	**0.454 [0.346, 0.595]**	**<0**.**001**
**2005–2009**	**0.416 [0.303, 0.571]**	**<0**.**001**
**≥2010**	**0.638 [0.409, 0.993]**	**0**.**046**
birth order	1.015 [0.926, 1.112]	0.755
older brother	0.970 [0.849, 1.108]	0.654
younger brother	1.022 [0.924, 1.131]	0.667
older sister	0.878 [0.760, 1.014]	0.076
younger sister	0.945 [0.841, 1.062]	0.342
residence husband's parents (n = 562)	**reference**	
with own parents (*n* = 726)	1.057 [0.863, 1.294]	0.592
with both parents (*n* = 832)	1.061 [0.862, 1.305]	0.575
**random effects**	**variance (s.d.)**	
mother ID	<0.001 (<0.001)	

### (c) Age at last birth (ALB)

The results of our ALB model can be seen in table 4. Contrary to predictions (table 1), women living in the same village as their own parents had an earlier age of last birth compared to women who resided in the same village as their husband's parents (HR = 1.392, figure 3*c*). In line with the AFB results above, residing with both parents was associated with an even higher hazard of ALB over time compared to women co-residing with just her husband's parents (HR = 1.688). Matching the IBI model, we find little evidence of associations with co-residence with brothers (table 4); however, co-residence with sisters was associated with a later ALB, a trend which was stronger for older sisters (HR = 0.758), suggestive of access to allomothers extending reproduction. In line with predictions (table 1), women in the reproductive cohorts after 1965 but prior to 2005 had increased hazards of stopping reproduction each year compared to those turning 16 prior to 1965, an effect largest in women reaching reproductive maturity in the 1980s whose reproductive careers completely overlapped with family planning policy implementation. Women starting reproduction after 2010 were not yet old enough to be considered as having had their last birth, hence their reduced hazard (HR = 0.08) of reproductive cessation (table 4).

**Table 4.** Cox proportional-hazards regression model of ALB (*n* = 963 females with 7765 person-years; having last birth = 1, not having last birth = 0, *n* = 817 events). Statistical significance is indicated in bold. Hazard ratios (HR) above 1.0 indicates earlier ALB.

variables	HR [95% CI]	*p*-value
cohort (ref: <1965)		
**1965–1969**	**2.057 [1.384, 3.058]**	**<0**.**001**
**1970–1974**	**2.580 [1.741, 3.824]**	**<0**.**001**
**1975–1979**	**4.430 [3.038, 6.458]**	**<0**.**001**
**1980–1984**	**6.505 [4.652, 9.096]**	**<0**.**001**
**1985–1989**	**8.005 [5.710, 11.222]**	**<0**.**001**
**1990–1994**	**5.450 [3.930, 7.558]**	**<0**.**001**
**1995–1999**	**5.666 [3.980, 8.067]**	**<0**.**001**
**2000–2004**	**2.529 [1.796, 3.562]**	**<0**.**001**
2005–2009	0.720 [0.445, 1.163]	0.179
**≥2010**	**0.080 [0.011, 0.582]**	**0**.**012**
birth order	1.090 [0.979, 1.215]	0.116
older brother	0.864 [0.741, 1.007]	0.062
younger brother	1.022 [0.908, 1.151]	0.716
**older sister**	**0.758 [0.642, 0.895]**	**0**.**001**
younger sister	0.917 [0.793, 1.060]	0.240
residence Husband's parents (*n* = 261)	**reference**	
**with own parents (*n* = 347)**	**1.392 [1.103, 1.757]**	**0**.**005**
**with both parents (*n* = 355)**	**1.688 [1.325, 2.150]**	**<0**.**001**

## 4. Discussion

Our analysis of over 1000 Tibetan women from Yunnan province, China, who have been reproductively active over the last 60 or more years reveals that co-residence with specific types of kin has associations with both AFB and ALB but not IBI. In this sample, compared to co-residing with only the husband's parents, co-residence with the wife's parents was associated with earlier AFB and ALB, while co-residing with *both* sets of parents predicted still earlier ages. More mixed results were found in relation to co-residence with a woman's siblings, with older siblings associated with later reproductive commencement and older sisters with later age at last birth. The largest observed effects were associated with the timing of reproductive maturity in relation to family planning policy implementation, which broadly predicted earlier AFB and ALB among those maturing in the 1980s and 1990s. These results are in line with a picture suggesting both the cooperative childrearing and sibling competition retain relevance for women's fitness, against a backdrop in which all women adaptively bring forward reproduction in response to caps on reproduction attenuating quality–quantity trade-offs.

### (a) Cooperative childrearing and AFB

The simplest form of the cooperative childrearing hypothesis states that individuals with more access to allomaternal investments have the *ability to* reproduce earlier, quicker and continue later because their energetic constraints have been lifted with the receipt of additional support [11,23,25]. Due to increased inclusive fitness returns, maternal kin are expected to provide more investments than paternal kin, thus this functional explanation may underpin our findings regarding parental co-residence and AFB. Similar results have been previously documented among the Mosuo, another ethnic minority within China, where AFB was found to be earlier in matrilocal versus patrilocal communities [50]. However, the cultural context does vary from that of the Mosuo, impacting the relationships between co-residence and fertility. The Mosuo are broadly matrilineal and duolocal but with some patrilocal villages [77]. In our field site, in contrast, post-marital residence is best understood as ambilocal in which couples may live in the same village with either of their parents, both or none. Such flexibility allows couples to reside with both sets of parents within the natal village, maximizing access to resources and allomaternal support across both kin groups. Accordingly, our results indicate that co-residence in the same village as both sets of parents is associated with the earliest AFB.

### (b) Conflict and reproductive timing

While the results for AFB are supportive of the cooperative childrearing hypothesis, the explanation of our ALB findings requires considering kin competition, as we find reproduction stops earlier, not later with increased access to potential grandparental allomothers. Such a finding also offers little support for the competition-based reproductive overlap model which predicts an earlier ALB in *patrilocal* settings based on shifting relatedness to the group over the life course [61], adding to earlier suggestions that this model has limited support in humans [11,62,78]. This is not a surprising finding for this sample; given the current low fertility of the population there is limited reproductive conflict between generations, reducing the indirect fitness pay-offs of earlier cessation of reproduction. Earlier ALB, within the context of restricted fertility, may be understood as a by-product of earlier AFB [11]. Given the government-mandated completed family size, the earlier couples commenced with reproduction, the earlier they will cease. This interpretation is supported (but not definitively so) by the uniform declines in AFB and ALB across time.

Parents are not the only co-residing relatives with which to potentially compete. Our analyses also explored the consequences of co-residence with the wife's siblings to elucidate if there is evidence of cooperative or conflictual relationships *within* generations. We found that AFB was increased when older siblings irrespective of sex were present in the same village. This relationship may be the product of younger siblings delaying their reproduction to act as ‘helpers-in-the-nest’ for their older siblings [9,48,58,79,80], demonstrating how cooperative childrearing can result in conflict. Certainly, previous studies have found similar effects whereby younger siblings show reproductive delays in a range of socio-ecological contexts [34,48,56,58]. Conflict can also be expressed in the optimal division of finite family resources. For instance, in this context, older daughters who remain at home inherit the family wealth, a major form of parental investment in their daughter's fitness [68]. In this context, younger siblings may delay and wait to begin reproduction, creating later ages for first and last birth when they are co-resident with older siblings. Indeed, competition for resources with siblings may favour flexibility in post-marital residential decision making to maximize compensatory access to parental resources. An alternative, though not mutually exclusive explanation to helping at the nest is that younger siblings are less competitive in the marriage market due to reduced parental investment and so their reproduction is delayed due to marriage delays. However, superficially pointing against this there is no difference by birth order both in the likelihood of marrying exogamously (influencing access to allocare) or age at marriage, and only 2.4% of the sample did not reproduce (see electronic supplementary material, table S8 and figure S3 for details). Conversely, we also found that ALB was later in association with co-residence with a woman's female siblings; this may suggest increased access to allocare facilitates continued reproduction.

### (c) IBIs and co-residence with kin

Unlike age at first and last birth, we see relatively little change in the length of interbirth intervals with different types of residence pattern, suggesting that once reproduction has begun, kin have little influence on its speed in this context. This is in opposition to data from Thailand [51], Indonesia [78], and historical Finland and Canada [42] in which co-residence with various grandparents (dependent on study context) was associated with shorter interbirth intervals. It may be that in the context of constrained completed fertility, quality–quantity trade-offs are relaxed to the extent that allomaternal support does not drive variation in optimal birth spacing. However, it is also important to note that 74% of mothers in this study had only two births, so most observed IBIs reflect the parallel shifts in timing of first and last birth.

### (d) Temporal changes in reproductive timing and the Fisherian effect

Finally, our results highlight large temporal trends which have occurred in Tibetan reproductive scheduling in the last 60 years. Hazard ratios for ALB were already increasing prior to childbearing restrictions being imposed in the early 1980s, indicating earlier reproductive cessation and likely lower completed fertility in those reaching reproductive maturity after 1965 compared to earlier. However, a sharper decline is seen in the 1970–79 cohort who were in their twenties when fertility was capped, suggesting a swift impact. ALB continued to decline among those maturing in the 1980s and plateaued in the 1990s cohorts, before increasing in those maturing in the 2000s. This ALB pattern broadly mirrors that documented by Mattison *et al*. [11] in the Mosuo, who experienced similar fertility regulation by Chinese authorities. Similarly, already secularly declining AFBs sharply declined in the early 1980's cohort, remaining at a consistently lower slightly fluctuating plateau in later cohorts. IBIs contract from the 1985–1989 to 2005–2009 cohort, largely as a product of age at second (and often last birth in this sample) birth falling at a greater rate than AFB.

These shifts to earlier reproductive timing in women whose twenties fell predominantly in the 1980s and 1990s, at a time when family planning policies were first introduced and later more strictly enforced, fit with a Fisherian adaptive response. Investment in reproduction earlier in life in a growing population with weak quality–quantity trade-offs (a likely result of upper fertility limits) is expected to be associated with higher fitness pay-offs because it shortens generational time, thus individuals who reproduce earlier represent a higher relative proportion of the gene pool than those who reproduce later [63,65,66]. Since fitness is always relative, timing matters. Deviations from this trend appears in those maturing after the 2000s with rising ALB, which may be the product of sociocultural shifts in the local ecology strengthening quality–quantity trade-offs. For example, compulsory education policy was implemented in 2000, meaning children began to receive formal education until middle school at higher rates [71], this may have raised pay-offs to delaying parity progression to maximize child ‘quality’.

A key limitation is that we do not have behavioural data to support the argument that grandparents of either lineage are providing allomaternal support, or for the cooperative and conflictual relationships between competitive behaviours of co-resident siblings. Allomaternal investments are only one factor among many which can influence female fertility. The consequences of co-residence are likely dependent on context-specific factors, such as distance between villages in which different kin reside and the possibility of kin of either lineage to travel and provide additional support, regardless of actual post-marital residence choice [20,81]. Consequently, further investigation into mechanisms associated with village co-residence, including assessing the relative importance of wealth inheritance and alloparenting, would be informative. More detailed data on the relative marriage prospects of earlier versus later born siblings, for instance whether younger siblings typically marry husbands of lower status, is also needed to be able to tease apart explanations for delayed AFB based on allocare and curtailed reproductive opportunities.

## 5. Conclusion

Our findings highlight a clear relationship between varying modes of post-marital residence and reproductive timing in a Tibetan population undergoing the demographic transition in the context of strict limitations on completed family sizes. We find evidence for both cooperation and conflict between co-resident kin, in line with the expectation that cooperative childrearing systems produce conflict in who gets to reproduce, and who supports that reproduction. In line with predictions, compared to women residing with only their husband's family, women living with just their own parents, or both sets of parents start reproduction earlier, likely resulting in earlier reproductive cessation. Co-resident siblings on the other hand were associated with delays in reproduction, highlighting competition over resources. These relationships are clear against a backdrop of large-scale temporal changes in reproductive timing in response to government-imposed constraints on completed fertility, in line with Fisherian expectations of fitness maximization when maximal fertility is capped. Ultimately, kin are associated with both direct fitness costs and benefits, particularly in a highly cooperative species like our own.

## Data Availability

Data files and code used to analyse and generate the figures are provided on the Open Science Framework: https://osf.io/sakq9/?view_only=b41a46e5b44b48c0a69e88f422b612f9. The data are provided in electronic supplementary material [82].
